# Ocular Telangiectasia and Cerebellar Atrophy in Ataxia-Telangiectasia (Louis-Bar Syndrome)

**DOI:** 10.5334/tohm.992

**Published:** 2025-01-20

**Authors:** Lukas Gattermeyer-Kell, Daniela Kern, Mariella Kögl, Petra Schwingenschuh

**Affiliations:** 1 Department of Neurology, Medical University of Graz, Graz, Austria

**Keywords:** Ataxia-telangiectasia, Louis-Bar syndrome, ocular telangiectasias, cerebellar atrophy, DNA repair, malignancies

## Abstract

**Background:**

Ataxia-telangiectasia (Louis-Bar syndrome) is a rare genetic disorder characterized by progressive ataxia, ocular telangiectasias, immunodeficiency and increased cancer risk due to impaired DNA repair.

**Phenomenology shown:**

Thorough clinical and subsequently radiological examination in a 19-year-old woman with a history of previously undiagnosed, progressive gait ataxia since early childhood, diffuse large B-cell lymphoma and severe combined immunodeficiency revealed the eponymous features of the disease, ocular telangiectasias and cerebellar atrophy, enabling targeted genetic testing.

**Educational value:**

Ocular telangiectasias represent an important clue for a diagnosis of ataxia-telangiectasia in young patients with progressive ataxia, implicating awareness of increased malignancy risk and treatment of immunodeficiency.

**Highlights:**

Ataxia-telangiectasia is a rare genetic disorder characterized by its eponymous features, progressive cerebellar ataxia and ocular telangiectasias. These signs can help in establishing an early diagnosis, hence preventing, or addressing secondary complications of the disease caused by impaired DNA repair, such as malignancies, immunodeficiency, and increased radiation sensitivity.

A 19-year-old woman presented with a history of progressive dystonic and ataxic gait disorder of unknown etiology since early childhood. History was significant for diffuse large B-cell lymphoma and severe combined immunodeficiency. The patient had one healthy younger sister and another sister suffering from similar neurologic symptoms. Both parents were healthy and there was no consanguinity.

Neurological examination was remarkable for oculomotor apraxia, scanning dysarthria, severe ataxia of the limbs, ataxia of stance and gait, and dystonic posturing of the right arm and leg. Cognition was unimpaired. Inspection of the eyes revealed ocular telangiectasias (Figure, Panel A). Cerebral magnetic resonance imaging (MRI) showed marked cerebellar, particularly vermal, atrophy (Figure, Panel B). Serum alpha-fetoprotein (AFP) level was considerably increased. Single gene sequencing of the *ATM*-gene (ataxia telangiectasia, mutated) uncovered a compound heterozygous mutation status (p.Tyr1284GInfsX9 in exon 28, p.Arg2032Lys in exon 43) in both affected siblings, confirming a diagnosis of ataxia-telangiectasia (Louis-Bar syndrome). Parental segregation analysis revealed a heterozygous mutation status of the *ATM*-gene in the patient’s mother (p.Tyr1284GInfsX9) and father (p.Arg2032Lys), respectively.

**Figure:**
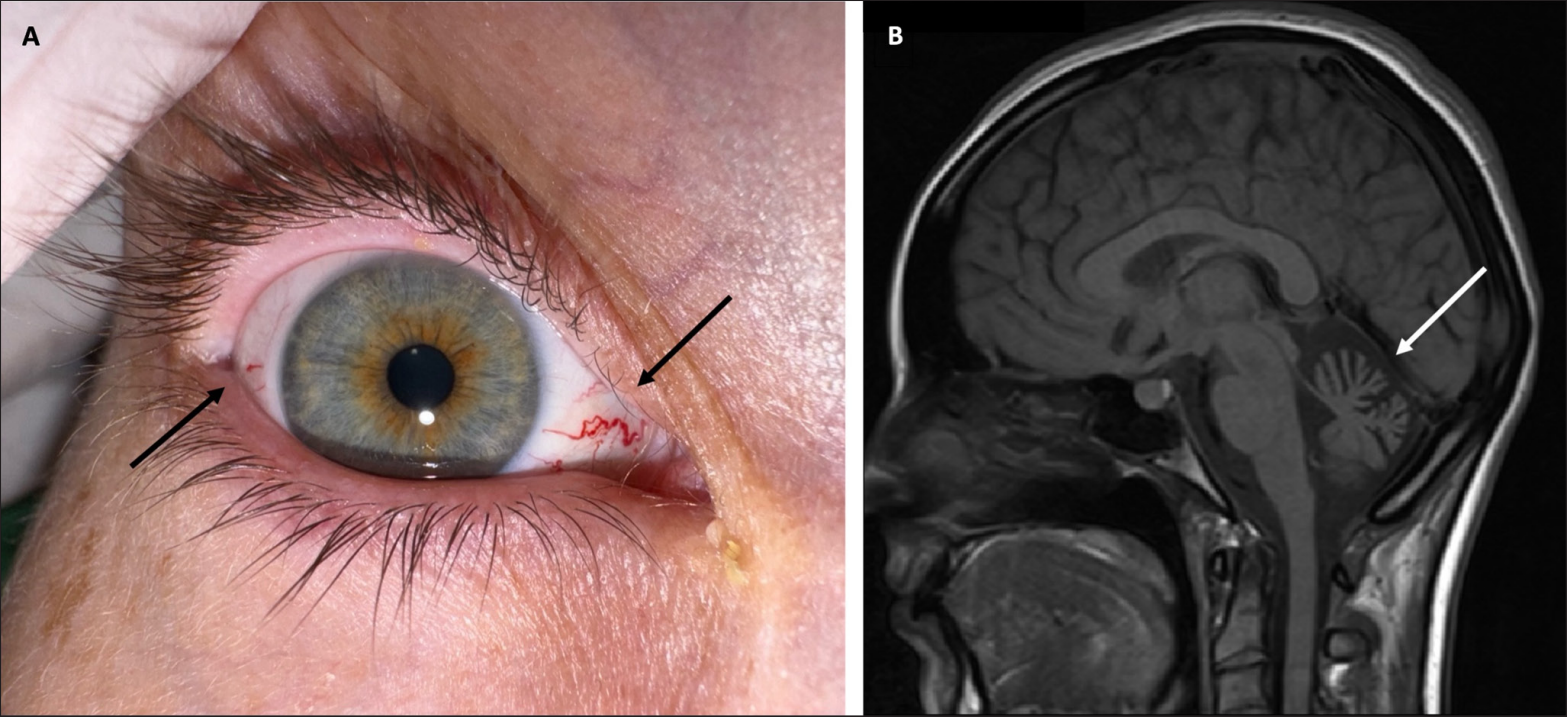
Ocular telangiectasias and cerebellar atrophy. Ocular telangiectasias (black arrows) in the patient’s right eye **(A)** and atrophy of the cerebellar vermis (white arrow) in T1-weighted sagittal cerebral magnetic resonance imaging **(B)**, representing the eponymous features of ataxia-telangiectasia - small, dilated blood vessels in the sclera and ataxia due to progressive cerebellar degeneration.

Immunodeficiency was treated with regular subcutaneous immunoglobulin injections. Neurologic deficits slowly progressed at follow-up visits.

Ataxia-telangiectasia is an autosomal recessive, progressive neurodegenerative disorder, hallmarked by increased cancer risk and radiation sensitivity due to impaired DNA repair, immunodeficiency, progressive ataxia, and ocular telangiectasias. Neuroimaging is characterized by cerebellar atrophy; serum AFP is often elevated, although the cause of this laboratory anomaly remains unclear. The diagnosis is confirmed by genetic testing [1]. While neurologic symptoms cannot be addressed causally, immunodeficiency, increased cancer risk and radiation sensitivity need to be treated or considered accordingly [1].

This case highlights the importance of thorough physical examination to guide targeted genetic testing and establish correct diagnoses in progressive neurological childhood disorders like ataxia-telangiectasia, helping to prevent its secondary complications.

## Abbreviations

MRI, magnetic resonance imaging; AFP, alpha-fetoprotein; ATM, ataxia telangiectasia, mutated; DNA, deoxyribonucleic acid.
